# Delayed blastulation and pregnancy outcomes in single frozen-thawed euploid blastocyst transfer

**DOI:** 10.3389/fendo.2025.1686274

**Published:** 2025-12-04

**Authors:** Jingwen Lang, Ling Hong, Wanli Yang, Qiaoling Wang, Yunqing Zhi, Xiuxian Zhu, Yonglun Fu

**Affiliations:** 1 Department of Assisted Reproductive Medicine, Shanghai First Maternity and Infant Hospital, School of Medicine, Tongji University, Shanghai, China; 2 Shanghai Key Laboratory of Maternal Fetal Medicine, Shanghai Institute of Maternal-Fetal Medicine and Gynecologic Oncology, Shanghai First Maternity and Infant Hospital, School of Medicine, Tongji University, Shanghai,, China

**Keywords:** euploid blastocyst transfer, delayed blastulation, frozen-thawed embryo transfer, embryo development speed, pregnancy outcomes, advanced maternal age

## Abstract

**Objective:**

This study aimed to evaluate the impact of embryo developmental speed on clinical outcomes in euploid blastocyst transfers, with a focus on maternal age and embryo morphological quality.

**Methods:**

A retrospective cohort study was conducted, including patients who underwent single blastocyst transfer following preimplantation genetic testing. Embryos were categorized based on developmental speed (day 5 *vs*. day 6 blastocysts) and further stratified by maternal age and embryo morphological quality. Clinical outcomes were compared between groups.

**Results:**

Day 5 euploid blastocysts yielded significantly higher implantation (68.8% *vs*. 48.3%) and live birth rates (63.7% *vs*. 40.4%) than day 6 blastocysts. In the advanced-age group, day 5 euploid embryos demonstrated significantly higher implantation (72.5% *vs*. 40.5%) and live birth rates (68.8% *vs*. 28.6%) than day 6 embryos. Developmental speed influenced pregnancy outcomes in low-quality embryos, where day 6 blastocysts exhibited a reduced live birth rate (29.3% *vs*. 65.5%) than day 5 blastocysts.

**Conclusion:**

Our findings indicate that day 5 euploid embryos are associated with better pregnancy outcomes compared to day 6 euploid embryos, with this trend being more pronounced in older participants and lower-quality embryo groups. However, day 6 euploid blastocyst transfer also yields acceptable implantation and pregnancy outcomes.

## What does this study add to the clinical work?

This study showed that day 5 euploid blastocysts are associated with superior pregnancy outcomes compared with day 6, particularly in older patients and those with lower-quality embryos. These findings can help assist in embryo selection during preimplantation genetic testing treatments.

## Introduction

Single-embryo transfer (SET) is increasingly recognized as an effective strategy to reduce the risks of multiple pregnancies and perinatal complications in assisted reproductive technology (ART) (1, 2). Selecting a single viable embryo is critical to reducing the time to pregnancy (3). Recently, advancements in embryo culture systems and incubation environments have facilitated the widespread adoption of blastocyst culture and transfer (4). Blastocyst transfer enhances embryo-endometrial synchronization and improves the likelihood of selecting viable embryos (5). Therefore, blastocyst transfer is considered a more physiologically optimal approach than cleavage-stage embryo transfer.

Beyond morphological quality, developmental speed is a key factor in selecting the most viable blastocysts. Studies indicate that transfer of day 5 blastocysts is associated with higher implantation and pregnancy rates compared to day 6 blastocysts (6, 7). However, other studies report no significant difference between these groups (8, 9). This inconsistency underscores the complexity of embryonic development and highlights the need for individualized treatment approaches. Recently, there has been growing interest in the potential of delayed-developmental embryos to optimize reproductive outcomes, particularly in patients with poor prognoses (10–12). A meta-analysis of retrospective cohort studies suggests that embryos with delayed blastulation may have lower live birth rates (LBRs) but should still be considered for clinical use (13).

Advancements in embryo assessment, including preimplantation genetic testing (PGT) for aneuploidy, have improved selection strategies by integrating genetic, morphological, and morphokinetic evaluations (14). Expanded blastocysts are associated with lower rates of aneuploidy compared to early-stage blastocysts (15, 16). Meanwhile, slower-developing embryos that reach the blastocyst stage on day 6 instead of day 5 have been linked to higher chromosomal abnormalities (17). This highlights the importance of evaluating slow blastulation embryos, particularly in patients undergoing PGT with a limited number of blastocysts available for transfer. Existing studies present conflicting evidence on whether embryo development speed affects pregnancy success following the transfer of a single euploid blastocyst (17, 18).

Given these considerations, we hypothesized that blastulation speed may reflect the developmental potential beyond ploidy. The aim of this study was to clarify the impact of slow blastulation on clinical outcomes following frozen embryo transfer (FET) by exclusively analyzing euploid embryos. Specifically, we sought to determine whether euploid day 6 blastocysts with slower developmental progress yield comparable clinical outcomes to euploid day 5 blastocysts. Additionally, stratified subgroup analyses were conducted, adjusting for maternal age and blastocyst quality while assessing developmental speed.

## Materials and methods

### Patient selection

This retrospective observational study was conducted between August 2020 and October 2024 at the Department of Shanghai First Maternity and Infant Hospital within the People’s Republic of China. The research investigators followed couples undergoing PGT combined with FET. The inclusion criteria were as follows: i) women aged 20–45 years; ii) PGT-A performed for advanced maternal age (≥ 38 years), recurrent pregnancy loss (≥2 consecutive pregnancy loss), or repeated implantation failure (≥4 embryos replaced or ≥2 blastocysts replaced without success); iii) PGT-SR performed for structural chromosomal rearrangements in either partner; iv) PGT-M performed for heritable monogenic conditions; and v) with at least one euploid blastocyst following biopsy. Participants were excluded based on any of the following: i) use of donor eggs or sperm; ii) presence of significant endometrial pathology, such as untreated hydrosalpinx or untreated endometrial polyps; and iii) endometrial thickness of <7 mm. Patients who failed to provide follow-up information were also excluded.

**Figure 1:**
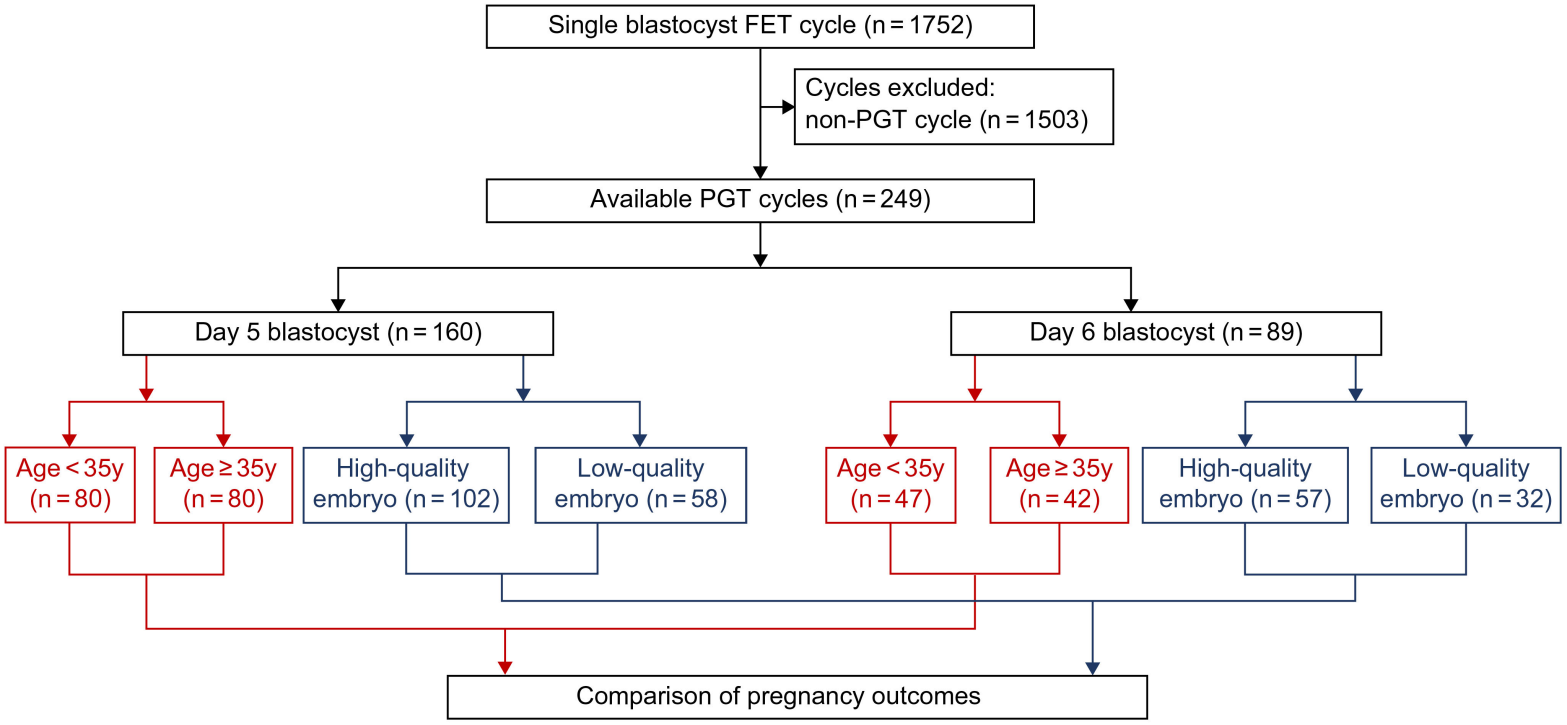
Flowchart of the study.

We included cycles involving two transfer strategies: infertile patients undergoing day 5 blastocyst transfer (*n* = 160) and patients undergoing day 6 blastocyst transfer (*n* = 89). Both groups were further stratified based on age and embryo quality. A visual flowchart in **Figure 1** illustrates the study design, comparing clinical outcomes from these FET approaches.

The present study was approved by the Research Ethics Committee of Shanghai First Maternity and Infant Hospital (approval no. #2023130) and was performed in accordance with the ethical standards as laid down in the 1964 Declaration of Helsinki and its later amendments or comparable ethical standards. The need for informed consent was waived due to the retrospective nature of this study.

### Blastocyst culture and scoring

Day 0 was defined as the day of oocyte retrieval. Intracytoplasmic sperm injection was performed, and viable blastocysts were cultured under conditions of 89% N_2_, 6% CO_2_, and 5% O_2_ post-fertilization. We assessed blastocyst development and morphology on days 5 and 6 and analyzed the embryos using the Gardner grading system (19). Blastocysts were first classified based on their degree of expansion:

Stage 1: Blastocoel occupying less than half of the volume.
Stage 2: Blastocoel occupying more than half of the volume.
Stage 3: Complete filling of the blastocoel within the embryo.
Stage 4: Expanded blastocyst with a thinning zona pellucida.
Stage 5: Beginning of hatching.
Stage 6: Fully hatched blastocyst.

Blastocysts at stages 3–6 were further evaluated for trophectoderm (TE) and inner cell mass (ICM) quality:

For TE grading: A (dense epithelial layer with numerous TE cells), B (sparse epithelial layer with fewer TE cells), or C (very sparse layer with few TE cells).
ICM grading: A (tightly packed, distinct cells), B (sparsely grouped cells), or C (small, indistinct cells).

Blastocysts with both ICM and TE grades of B or higher (≥3BB) were classified as high quality, whereas all others were considered low quality. Stage 3–6 blastocysts were cryopreserved.

### Blastocyst biopsy and euploidy diagnosis

Once fully expanded, blastocysts underwent biopsy following the method described by McArthur et al. (20). Hatched trophoblasts were stabilized using holding pipettes (Origio, Malov, Denmark) and aspirated with a biopsy needle (Vitrolife, Gothenburg, Sweden). Three to five trophoblast cells were collected using a combination of laser cutting and aspiration. For unhatched blastocysts, trophoblast cells were aspirated through an opening in the zona pellucida using a biopsy needle through negative pressure.

Extracted ectodermal trophoblast cells were lysed in 5 μL of KOH solution, followed by whole genome amplification. Chromosomal aneuploidy was assessed using a Human CytoSNP-12 DNA array (Illumina, San Diego, USA).

### Freezing and thawing protocol

Chromosomally normal blastocysts were preserved using the Kitazato vitrification system (Kitazato Biopharma, Fuji, Japan) and Kitazato vitrification straws (Kitazato Biopharma). A commercially available warming solution (Kitazato Biopharma) was used for thawing. Post-thawing, embryos were incubated in a Trigas incubator set at 37°C under 6% CO_2_, 5% O_2_, and 90% N_2_. Viable, intact embryos were identified as those that remained structurally intact and resumed development post-thawing.

### Endometrial preparation

Endometrial preparation was achieved using controlled ovarian stimulation (COS), natural cycle (NC), or hormone replacement therapy (HRT). In the COS and NC groups, follicular monitoring was performed on the fifth day following ovulation to confirm endometrial thickness >7 mm. In the artificial protocol, estradiol (6 mg orally) was administered continuously, and progesterone was introduced once the endometrial thickness exceeded 7 mm. Embryo transfer was scheduled 5 days later.

### Outcome measures and follow-up

The primary outcome measure was the live birth rate. The secondary outcome measures included the rates of clinical pregnancy, implantation, early/late miscarriage, and neonatal outcomes. Maternal serum human chorionic gonadotropin (hCG) levels were measured 12 days post-FET. Serum β-hCG levels were considered positive when they exceeded 20 IU/L, which was defined as a biochemical pregnancy. A transvaginal ultrasound on day 21 post-transfer confirmed clinical pregnancy by detecting a gestational sac. The implantation rate was calculated as the number of gestational sacs per number of transferred embryos. We determined the live birth rate by dividing the total number of live births by the total number of embryo transfer cycles. Patients with two or more gestational sacs were considered to have multiple conceptions. An early miscarriage was defined as the spontaneous termination of a pregnancy before the end of the third month. A late miscarriage was defined as a pregnancy ending after 12 weeks of gestation. A gestational sac that developed and settled outside the uterine endometrial cavity was considered an ectopic pregnancy. The multiple pregnancy rate was defined as the proportion of pregnancies with two or more fetuses. Gestational age was defined as the cumulative days during pregnancy. Babies weighing less than 2,500 g at birth were considered to have low birth weight (LBW). We considered deliveries before 37 weeks as preterm births. As part of regular follow-up examinations, researchers contacted couples by phone to collect neonatal data, such as due date, birth weight, height, sex, birth abnormalities, and newborn illnesses.

### Statistical analysis

The Kolmogorov–Smirnov test was used to assess data normality. Normally distributed continuous variables were presented as mean ± standard deviation (SD), whereas non-normally distributed data were expressed as median with interquartile range (IQR). Categorical variables were presented as frequencies and percentages.

Between-group differences were analyzed using independent *t*-tests for normally distributed continuous variables and the Mann–Whitney *U* test for non-normally distributed data. The chi-square test was used to compare between-group categorical variables, with Fisher’s exact test applied if expected frequencies were <5.

Logistic regression analysis was performed to adjust for confounding factors, including advanced age (≥35 years), body mass index (BMI), anti-Müllerian hormone (AMH) levels, duration of infertility, embryo transfer timing, endometrial thickness, and blastocyst quality. Statistical significance was defined as *P* <0.05. All statistical analyses were performed using the Statistical Package for Social Sciences (SPSS) version 27. 0 for Windows (IBM Corporation, Armonk, USA).

## Results

### Patient characteristics

The study incorporated 249 PGT–FET cycles (**Table 1**). Patients who underwent PGT–FET were divided into a day 5 blastocyst transfer group (day 5, *n* = 160) and a day 6 blastocyst transfer group (day 6, *n* = 89). In total, 249 embryos were transferred. Statistical analysis showed no significant differences between day 5 and day 6 groups in maternal age, BMI, AMH level, menstrual cycle, type of infertility, duration of infertility, etiology, time of embryo transfer, endometrial preparation, endometrial thickness, or the rate of high-quality embryos.

**Table 1:** Baseline characteristics of patients in the day 5 and day 6 groups.

Characteristics	PGT cycles		*P*
	Day 5	Day 6	
Cycles (*n*)	160	89	
Maternal age (years) (mean ± SD)	34.98 ± 4.76	35.02 ± 4.54	0.947
BMI (kg/m^2^) (mean ± SD)	21.66 ± 3.18	22.17 ± 3.17	0.223
AMH (ng/mL) (mean ± SD)	4.50 ± 2.95	3.76 ± 3.01	0.098
Menstrual cycle, *n* (%)			0.295
Regular	124 (77.5)	73 (81.1)	
Irregular	36 (22.5)	17 (18.9)	
Type of infertility, *n* (%)			0.586
Primary	33 (20.6)	21 (23.6)	
Secondary	127 (79.4)	68 (76.4)	
Duration of infertility (years) (IQR)	1.55 (2)	1.4 (2)	0.935
Infertile etiology, *n* (%)			0.450
Female	130 (81.3)	76 (85.4)	
Male	20 (12.5)	7 (7.9)	
Combination	9 (5.6)	4 (4.5)	
Uncertain	1 (0.6)	2 (2.2)	
Time of embryo transfer, *n* (%)	1 (1)	1 (2)	0.241
Endometrial preparation, *n* (%)			0.659
COS	120 (75.0)	65 (73.0)	
NC	19 (11.9)	14 (15.7)	
HRT	21 (13.1)	10 (11.2)	
Endometrial thickness (mm) (IQR)	10.65 (3.5)	10.20 (4.1)	0.445
High-quality embryo, *n* (%)	102 (63.7)	48 (53.9)	0.129

Day 5, day 5 blastocyst group; day 6, day 6 blastocyst group; AMH, anti-Müllerian hormone; COS, controlled ovarian stimulation; NC, natural cycle; HRT, hormone replacement treatment; IQR, interquartile range; PGT, preimplantation genetic testing; BMI, body mass index.

### Clinical outcomes

**Figure 2:**
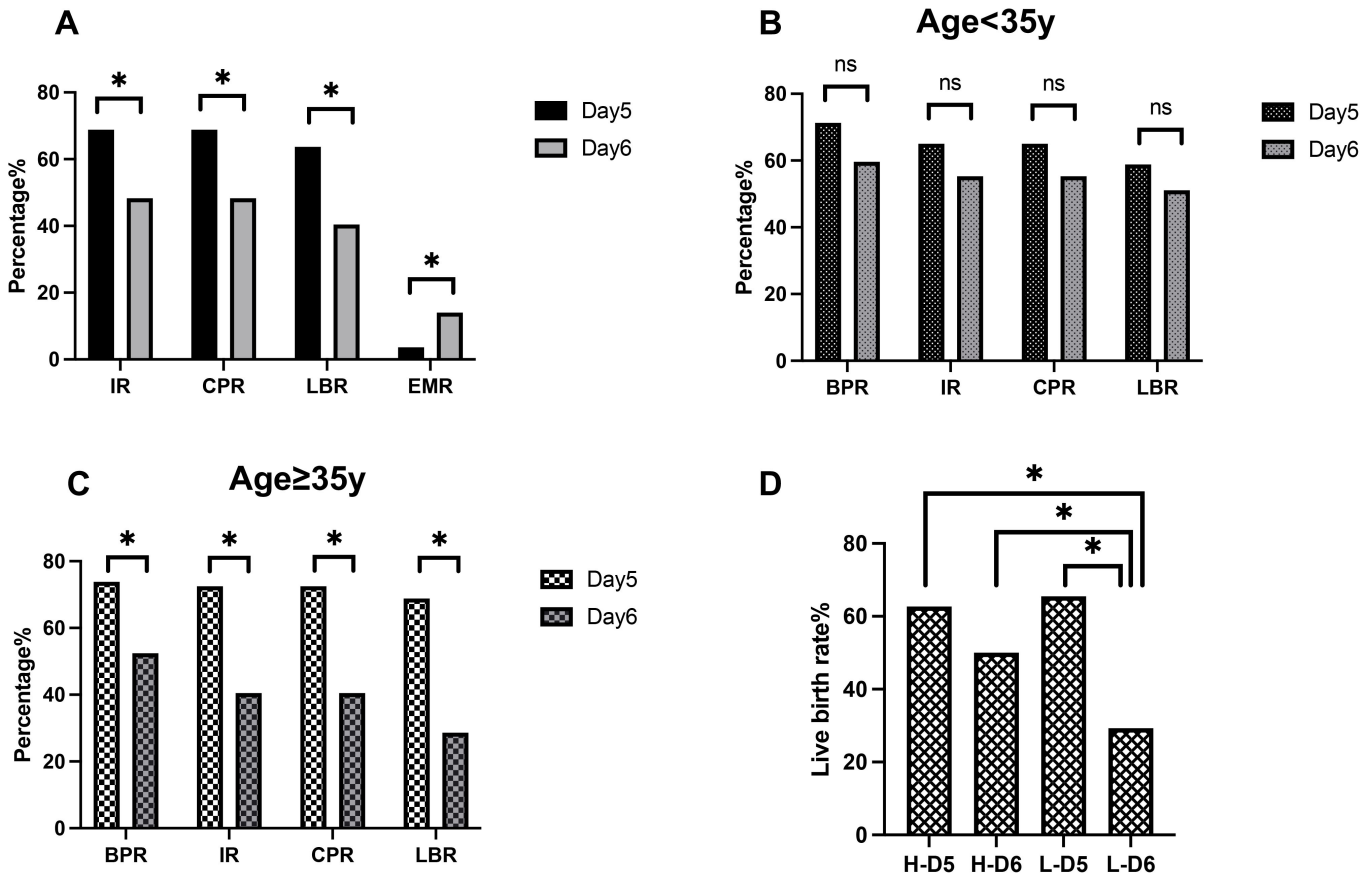
Relationship between blastulation speed, maternal age, embryo quality, and clinical pregnancy outcomes. **(A)** Pregnancy outcomes of blastocysts. **(B)** Pregnancy outcomes of patients with a younger age. **(C)** Pregnancy outcomes in patients with advanced age. **(D)** Live birth rates in the high-quality and low-quality embryo groups. IR, implantation rate; BPR, biochemical pregnancy rate; CPR, clinical pregnancy rate; LBR, live birth rate; EMR, early miscarriage rate; H-D5, high-quality day 5 embryos; H-D6, high-quality day 6 embryos; L-D5, low-quality day 5 embryos; L-D6, low-quality day 6 embryos. *P < 0.05.

The pregnancy, delivery, and neonatal outcomes of both groups are presented in **Table 2**. The rates of biochemical pregnancy (72.5% *vs*. 56.2%, *P* = 0.009), implantation (68.8% *vs*. 48.3%, *P* = 0.001), clinical pregnancy (68.8% *vs*. 48.3%, *P* = 0.001), and live birth (63.7% *vs*. 40.4%, *P* < 0.001) were significantly higher in the day 5 group compared to the day 6 group (**Figure 2A**). The early miscarriage rate was lower in the day 5 group than in the day 6 group (3.6% *vs*. 14.0%, *P* = 0.030) (**Figure 2A**). However, there were no significant differences in the rates of ectopic pregnancy, multiple pregnancies, or late miscarriage between the two groups. Regarding delivery and neonatal outcomes, metrics including gestational age, preterm birth rate, neonatal sex distribution, birth weight, birth height, low birth weight, and birth defects were all comparable between the day 5 and day 6 blastocyst groups.

**Table 2:** Pregnancy, delivery, and neonatal outcomes in the day 5 and day 6 groups.

Outcomes	PGT cycles		*P*
	Day 5	Day 6	
Pregnancy outcomes			
*n*	160	89	
Biochemical pregnancy, *n* (%)	116 (72.5)	50 (56.2)	0.009*
Clinical pregnancy, *n* (%)	110 (68.8)	43 (48.3)	0.001*
Implantation, *n* (%)	110 (68.8)	43 (48.3)	0.001*
Multiple pregnancy, *n* (%)	2 (1.8)	1 (2.3)	1.000
Early miscarriage, *n* (%)	4 (3.6)	6 (14.0)	0.030*
Ectopic pregnancy, *n* (%)	1 (0.9)	0 (0)	1.000
Late miscarriage, *n* (%)	3 (1.9)	1 (1.1)	0.651
Live birth, *n* (%)	102 (63.7)	36 (40.4)	<0.001*
Delivery outcomes			
Gestational age (days) (IQR)	272 (8)	273 (9)	0.322
Preterm birth, *n* (%)	6 (5.9)	3 (8.3)	0.609
Neonatal outcomes			
Male neonates, *n* (%)	59 (56.7)	19 (52.8)	0.681
Female neonates, *n* (%)	45 (43.3)	17 (47.2)	
Birth weight (g) (mean ± SD)	3,209.5 ± 448.8	3,230.7 ± 535.1	0.509
Birth height (cm) (mean ± SD)	49.7 ± 1.4	49.6 ± 2.0	0.380
Low birth weight, *n* (%)	7 (6.7)	2 (5.6)	0.804
Birth defects, *n* (%)	2 (1.9)	0 (0)	1.000

Day 5, day 5 blastocyst group; day 6, day 6 blastocyst group; hCG, human chorionic gonadotropin; PGT, preimplantation genetic testing.

*P < 0.05.

A subgroup analysis based on maternal age revealed similar trends (**Table 3**). Among women of advanced maternal age(≥35 years old), the rates of biochemical pregnancy (73.8% *vs*. 52.4%, *P* = 0.018), implantation (72.5% *vs*. 40.5%, *P* = 0.001), clinical pregnancy (72.5% *vs*. 40.5%, *P* = 0.001), and live birth (68.8% *vs*. 28.6%, *P* < 0.001) were significantly higher in the day 5 group than in the day 6 group (**Figure 2C**). Additionally, the early miscarriage rate was lower in the day 5 group (3.4% *vs*. 23.5%, *P* = 0.021). However, in younger women (<35 years old), these trends did not reach statistical significance (**Figure 2B**).

**Table 3:** Comparison of clinical outcomes between day 5 and day 6 in young and advanced-age patients undergoing preimplantation genetic testing–frozen embryo transfer (PGT–FET).

Clinical outcomes	Age <35 years		*P*	Age ≥35 years		*P*
	Day 5	Day 6		Day 5	Day 6	
n	80	47		80	42	
Biochemical pregnancy, *n* (%)	57 (71.3)	28 (59.6)	0.177	59 (73.8)	22 (52.4)	0.018*
Clinical pregnancy, *n* (%)	52 (65.0)	26 (55.3)	0.279	58 (72.5)	17 (40.5)	0.001*
Implantation, *n* (%)	52 (65.0)	26 (55.3)	0.279	58 (72.5)	17 (40.5)	0.001*
Early miscarriage, *n* (%)	2 (3.8)	2 (7.7)	0.468	2 (3.4)	4 (23.5)	0.021*
Live birth, *n* (%)	47 (58.8)	24 (51.1)	0.400	55 (68.8)	12 (28.6)	<0.001*

Day 5, day 5 blastocyst group; day 6, day 6 blastocyst group.

*P < 0.05.

### Embryo quality and live birth

To assess the impact of embryo quality on live birth rates, all 249 cycles were categorized into four groups: high-quality day 5 embryos (H-D5), high-quality day 6 embryos (H-D6), low-quality day 5 embryos (L-D5), and low-quality day 6 embryos (L-D6).

As shown in **Table 4** and **Figure 2D**, regardless of embryo quality, live birth rates were higher among day 5 transfers than in day 6 transfers (H-D5: 62.7% *vs*. H-D6: 50.0%, L-D5: 65.5% *vs*. L-D6: 29.3%). This difference was statistically significant in the low-quality embryo groups (*P* < 0.001).

**Table 4:** *P*-values of *χ*^2^ analysis for live birth rate between the day 5 and day 6 groups in patients with high- and low-quality embryos undergoing preimplantation genetic testing–frozen embryo transfer (PGT–FET).

Group (*N*) (live birth rate %)	H-D5 (*n* = 102)	H-D6 (*n* = 58)	L-D5 (*n* = 48)	L-D6 (*n* = 41)
H-D5 (62.7%)	–	0.139	0.726	<0.001*
H-D6 (50.0%)	0.139	–	0.107	0.047*
L-D5 (65.5%)	0.726	0.107	–	<0.001*
L-D6 (29.3%)	<0.001*	0.047*	<0.001*	–

H-D5, day 5 high-quality blastocyst; H-D6, day 6 high-quality blastocyst; L-D5, day 5 low-quality blastocyst; L-D6, day 6 low-quality blastocyst.

*P < 0.05.

Within the day 5 group, live birth rates were comparable between high-quality and low-quality embryos (62.7% *vs*. 65.5%, *P* = 0.726). In contrast, in the day 6 group, the high-quality group had a higher ongoing pregnancy rate than the low-quality group (50.0% *vs*. 29.3%, *P* = 0.047). The live birth rate in the low-quality day 5 group (65.5%) was over 15% higher than in the high-quality day 6 group (50.0%), although this difference did not reach statistical significance (*P* = 0.107).

### Logistic regression analysis

A logistic regression analysis was performed to compare live birth rates per transfer cycle between day 5 and day 6 (**Table 5**). In the unadjusted model, the likelihood of live birth was significantly higher in day 5 transfers than in day 6 [odds ratio (OR) = 2.59, 95% confidence interval (CI): 1.52–4.41]. After adjusting for potential confounders [maternal advanced age (≥35 years/<35 years), BMI, AMH level, duration of infertility, time of embryo transfer, endometrial thickness, and quality of embryos], this association remained significant [adjusted odds ratio (AOR) = 3.32, 95% CI: 1.74–6.34]. Additionally, a lower BMI was significantly associated with a higher live birth rate (AOR = 0.90, 95% CI: 0.82–0.99).

**Table 5:** Logistic regression analysis for live birth.

Parameter	Unadjusted		Adjusted	
	OR (95% CI)	*P*	AOR (95% CI)	*P*
PGT (day 5/day 6)	2.59 (1.52–4.41)	<0.001*	3.32 (1.74–6.34)	<0.001*
Age (≥35 years/<35 years)	–	–	0.83 (0.42–1.64)	0.600
BMI	–	–	0.90 (0.82–0.99)	0.032*
AMH	–	–	1.07 (0.96–1.20)	0.227
Time of embryo transfer	–	–	1.01 (0.84–1.22)	0.937
Duration of infertility	–	–	0.91 (0.79–1.06)	0.214
Endometrial thickness	–	–	1.07 (0.96–1.20)	0.202
High-quality embryo	–	–	1.73 (0.92–3.28)	0.092

Day 5, day 5 blastocyst group; day 6, day 6 blastocyst group; BMI, body mass index; AMH, anti-Müllerian hormone; OR, odds ratio; AOR, adjusted odds ratio; CI, confidence interval.

*P < 0.05.

## Discussion

In this retrospective cohort study, we evaluated the impact of slow blastulation (day 6 blastocyst) on pregnancy outcomes following FET using only euploid embryos. Our findings demonstrated a significant decrease in live birth rates and a notable increase in early miscarriage risk in day 6 blastocyst transfers compared to day 5. This trend was more pronounced in women of advanced age and patients with low-quality blastocysts. Logistic regression analysis further confirmed the advantage of day 5 blastocyst transfer over day 6 in terms of live birth rates. Our study provides clear evidence that, even among euploid embryos, the developmental potential of a blastocyst is significantly influenced by its speed of formation.

Despite the significant improvement in implantation rates with PGT, a considerable proportion of euploid embryos still fail to implant or result in miscarriage (21). In a retrospective study, laboratory data and clinical outcomes from 1,051 single euploid embryo FET cycles were analyzed. Women were categorized into two groups based on pregnancy outcomes: live births and miscarriages. Although the ICM and trophectoderm scores were not significantly different, the percentage of embryos biopsied on day 6 was significantly higher in the miscarriage group (22). Another study also revealed a trend toward higher miscarriage rates in day 6 euploid embryos compared to day 5 (13.33% *vs*. 9.20%) in women who underwent PGT (18). In the present study, the early miscarriage rate was significantly lower in the day 5 group than in the day 6 group (3.6% *vs*. 14.0%, *P* = 0.030). However, among younger women, this trend was not statistically significant. These findings suggest that delayed development per se may be a manifestation of intrinsic embryonic deficiencies, such as impaired mitochondrial function leading to inadequate energy supply, or age-related accumulation of epigenetic errors. While these factors do not affect chromosomal ploidy, they may severely compromise the embryo’s post-implantation developmental potential, ultimately resulting in pregnancy loss.

Advanced age is associated with a decline in ovarian reserve and oocyte quality, both of which can impact embryonic development and pregnancy outcomes. A meta-analysis assessing the impact of PGT in different age groups found that PGT improved live birth in patients over 35 years old (OR = 1.29; 95% CI: 1.05–1.60; *n* = 692), whereas it had no effect in younger women (OR = 0.92; 95% CI: 0.62–1.39; *n* = 666) (23). Our subgroup analysis based on maternal age revealed a pronounced advantage for day 5 blastocysts, which demonstrated significantly higher rates of implantation (72.5% *vs*. 40.5%; *P* = 0.001) and live birth (68.8% *vs*. 28.6%; *P* < 0.001) over their day 6 counterparts. Additionally, the early miscarriage rate was significantly lower in day 5 transfers (3.4% *vs*. 23.5%, *P* = 0.021). With advanced maternal age, oocytes are more likely to exhibit mitochondrial dysfunction, resulting in inadequate adenosine triphosphate (ATP) production (24). A recent study analyzing uniformly biopsied euploid blastocysts revealed that mtDNA content is associated with developmental speed and morphological quality (25). A slower-developing day 6 blastocyst may represent an embryo with compromised energy metabolism, struggling to complete key developmental events in a timely manner. Furthermore, advanced age is associated with accumulated epigenetic alterations in oocytes, such as errors in DNA methylation imprinting (26). A delayed blastulation may be a visible manifestation of these irregularities, which can disrupt proper gene expression and lead to failure in implantation or miscarriage.

Developmental speed and morphological quality jointly reflect the developmental potential of the embryo, yet a key clinical dilemma persists: which parameter should carry greater weight in selection priority?

The study found no significant differences in pregnancy and implantation rates between high-quality day 5 and day 6 blastocysts (52.4% *vs*. 52.6% and 38.9% *vs*. 35.6%; *P* = 0.97 and *P* = 0.39, respectively). In contrast, low-quality day 6 blastocysts exhibited remarkably lower pregnancy and implantation rates than their day 5 counterparts (42.0% *vs*. 29.8% and 29.7% *vs*. 23.1%; *P* = 0.01 and *P* = 0.04, respectively) (27). In our day 5 group, live birth rates were nearly identical between high- and low-quality blastocysts (62.7% *vs*. 65.5%, *P* = 0.726). Conversely, in the day 6 group, the live birth rate was higher for high-quality blastocysts than for low-quality ones (50.0% *vs*. 29.3%, *P* = 0.047). These findings demonstrate that the impact of morphological quality on live birth rate is critical in slower-developing (day 6) blastocysts but less pronounced in faster-developing ones (day 5). Additionally, when comparing L-day 5 blastocysts with H-day 6 blastocysts, the live birth rate was approximately 15% higher in the day 5 group (65.5% *vs*. 50.0%), although statistical significance was not reached. This finding partially addresses a common clinical question: which has better developmental potential—a “4BB blastocyst on day 6” or a “4BC blastocyst on day 5”? The lack of statistical significance may be attributed to the relatively small sample size. For rapidly developing day 5 blastocysts, their robust intrinsic vitality compensates for morphological shortcomings, thereby diminishing the relative importance of morphology scores. Conversely, for slower-developing day 6 blastocysts, morphological grading becomes a critical tool for identifying the most viable embryos within a cohort of lower potential. Thus, the developmental speed of a blastocyst appears to reflect a superior underlying biological state that outweighs the significance of morphological grading, offering a decisive criterion for embryo selection. This underlying biological state likely involves metabolic efficiency, genomic integrity (beyond ploidy), and epigenetic homeostasis—all critical for timely development and successful implantation.

Our multivariate regression analysis confirmed that blastocyst development speed (day 5/day 6) remained significantly associated with live birth rates. This finding is consistent with a previous study (28). However, maternal age (≥35 years/<35 years) was not an independent predictor of live birth in our model. This result contrasts with some larger studies, which have identified advanced maternal age as a risk factor for inferior outcomes, even following the transfer of euploid embryos (29, 30). However, other studies found that this effect appears to be not statistically significant or pronounced only at the extremes of maternal age (e.g., ≥42 years) (28, 31). A potential explanation for this discrepancy lies in the detrimental effect of advanced age on compromising embryo competence, as reflected in poorer morphology and delayed development (31). Consequently, in our model that adjusted for embryo morphology and development speed—the very parameters through which age may exert its influence—the independent effect of age was likely attenuated. Furthermore, with a limited sample size [<35 (*n* = 127), 35–37 (*n* = 36), 38–40 (*n* = 45), 41–42 (*n* = 30), and >42 (*n* = 11)], our study was likely underpowered to detect this effect.

Our study provides evidence that supports the idea: delayed blastulation is an independent negative predictor of live birth in PGT–FET. The stratified analysis by maternal age suggests that advanced maternal age adversely affects reproductive outcomes through mechanisms independent of aneuploidy. Another strength of our study is the subgroup analysis by embryo morphology, which provides a data-driven reference for the embryo selection strategy considering both developmental speed and morphological grade.

However, several limitations must be considered. The retrospective nature of the study carries the risk of selection bias and unmeasured confounding, despite our statistical adjustments. The sample size, particularly in the day 6 subgroup (*n* = 89) and its further divisions (e.g., low-quality day 6), was relatively small. With a limited sample size, the subgroup analyses were exploratory and were not adjusted for confounders, and their results should be interpreted with caution. Furthermore, during the study period, two euploid blastocysts that reached the blastocyst stage on day 7 were also transferred. One of them resulted in a live birth. However, due to the extremely small sample size (*n* = 2), day 7 cases were not included in the formal statistical comparisons. Studies with larger samples of day 7 euploid blastocysts are warranted to definitively assess their developmental potential. Future research should incorporate larger cohorts and investigate molecular mechanisms, such as mitochondrial function and epigenetic regulation, to further elucidate these findings. Our study builds on existing evidence, clarifying the relationship between blastocyst developmental speed and implantation potential. These insights may aid in counseling patients undergoing PGT-SET to optimize blastocyst selection for improved clinical outcomes.

## Conclusion

Our findings indicate that day 5 euploid embryos are associated with better pregnancy outcomes compared with day 6 euploid embryos, with this trend being more pronounced in older participants and lower-quality embryo groups. However, single blastocyst transfer of day 6 euploid embryos also yields acceptable implantation and pregnancy outcomes. Future research should incorporate larger cohorts to further elucidate these findings and explore underlying biological mechanisms to enhance clinical outcomes and refine selection criteria.

## Data Availability

The raw data supporting the conclusions of this article will be made available by the authors, without undue reservation.
